# An unusual cause of a large fibrinous pericardial effusion

**DOI:** 10.5830/CVJA-2014-075

**Published:** 2015

**Authors:** Noleen Chengetai Tembani-Munyandu, Leolin Katsidzira, Rudo Makunike-Mutasa, Andrew Chinogureyi

**Affiliations:** Department of Medicine, College of Health Sciences, University of Zimbabwe, Harare, Zimbabwe; Department of Medicine, College of Health Sciences, University of Zimbabwe, Harare, Zimbabwe; Department of Histopathology, College of Health Sciences, University of Zimbabwe, Harare, Zimbabwe; Department of Radiology, College of Health Sciences, University of Zimbabwe, Harare, Zimbabwe

**Keywords:** fibrinous pericardial effusion, tuberculosis (TB), angiosarcoma

## Abstract

The commonest cause of a large fibrinous pericardial effusion in sub-Saharan Africa is tuberculosis. There are, however, limited resources available for making a definitive diagnosis of tuberculous pericarditis. The diagnosis is largely based on clinical criteria. There is a risk of misdiagnosing lesscommon causes of large fibrinous pericardial effusions. We present a patient who had a pericardial angiosarcoma that was initially thought to be a tuberculous pericardial effusion, and discuss the challenges in making a definitive diagnosis of tuberculosis.

## Abstract

The majority of fibrinous pericardial effusions in sub-Saharan Africa are caused by tuberculosis (TB), which accounts for such effusions in more than 80% of HIV-positive patients, and 50 to 70% of HIV-negative patients in the region.^1^-^4^ Consequently, it is a common clinical practice to commence patients on TB treatment on the basis of a fibrinous pericardial effusion seen on echocardiogram.

While this strategy may have merit, there is the risk that patients with less-common causes of a fibrinous effusion may have their diagnosis and treatment unduly delayed. We present a patient who had a large fibrinous pericardial effusion, which was managed as tuberculosis, but this turned out not to be the case.

## Case report

A 24-year-old male was referred from a peripheral hospital complaining of two months’ history of shortness of breath on exertion, left-sided pleuritic chest pain, a non-productive cough and significant weight loss. He had lost 17 kg over two months and had drenching night sweats.

He had drunk at least 40 units of alcohol per week and smoked two packs of cigarettes per week for a year. He had no significant medical history and had tested HIV negative a month prior to presentation.

On examination he was wasted, had no significant lymphadenopathy and had a temperature of 37.2°C. His blood pressure was 96/50 mmHg and he had a low-volume tachycardia of 116 beats per minute. The jugular venous pressure was 7 cm and the apex beat could not be localised. The heart sounds were muffled and he had a tender hepatomegaly 4 cm below the costal margin.

The chest X-ray showed a large globular heart shadow but no pulmonary congestion. The electrocardiogram showed a sinus tachycardia and echocardiography revealed a large fibrinous pericardial effusion about 5 cm in maximum depth. There was early right ventricular diastolic collapse and the inferior vena cava was 2.5 cm and not collapsing with inspiration.

The full blood count showed a white cell count of 6.4 × 10^3^ cells/μl, haemoglobin 14.6 g/dl and platelet count 275 × 10^3^ cells/μl. Urea and electrolytes were sodium 126 mmol/l, potassium 3.4 mmol/l, urea 9.1 mmol/l and creatinine 88 μmol/l. Serum albumin was 28 g/l, total protein was 81 g/l and serum globulin was 53 g/l.

A probable diagnosis of TB pericardial effusion was made on the basis of a Tygerberg score^5^ of 8: night sweats = 1, weight loss = 1, fever > 37.8°C = 0, white cell count < 10 cells/μl = 3, serum globulin > 40 g/l = 3, total score = 8.

The patient underwent urgent pericardiocentesis and 1 000 ml of haemorrhagic pericardial effusion were drained. The fluid microscopy showed a negative Ziehl Nielsen stain, a few leucoytes, many red blood cells, protein of 55 g/l, lactate dehydrogenase was 1 456 U/l and adenine deaminase was 24 U/l. He was commenced on TB therapy (isoniazid, rifampicin, pyrazinamide and ethambutol).

Despite treatment, he continued to have severe chest pain and progressive loss of weight. On review at six weeks, there was echocardiographic evidence of re-accumulation of the fluid and he was experiencing severe pain in the thoracic spine.

**Figure 1.**
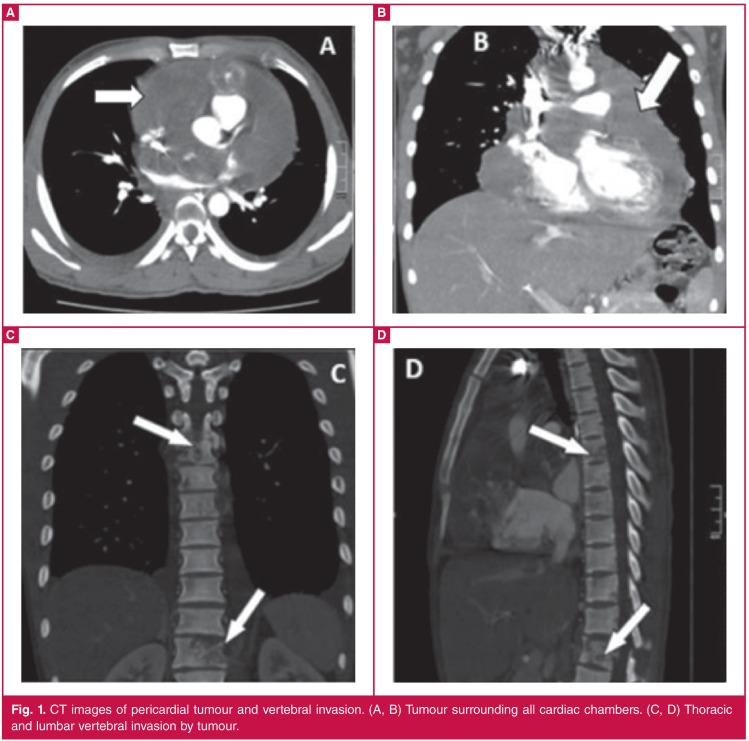
CT images of pericardial tumour and vertebral invasion. (A, B) Tumour surrounding all cardiac chambers. (C, D) Thoracic and lumbar vertebral invasion by tumour.

A computed tomography scan was requested and it showed a tumour in the pericardium compressing the cardiac chambers and extending posteriorly into the thoracic spine and upper lumbar spine (Fig. 1).

**Figure 2.**
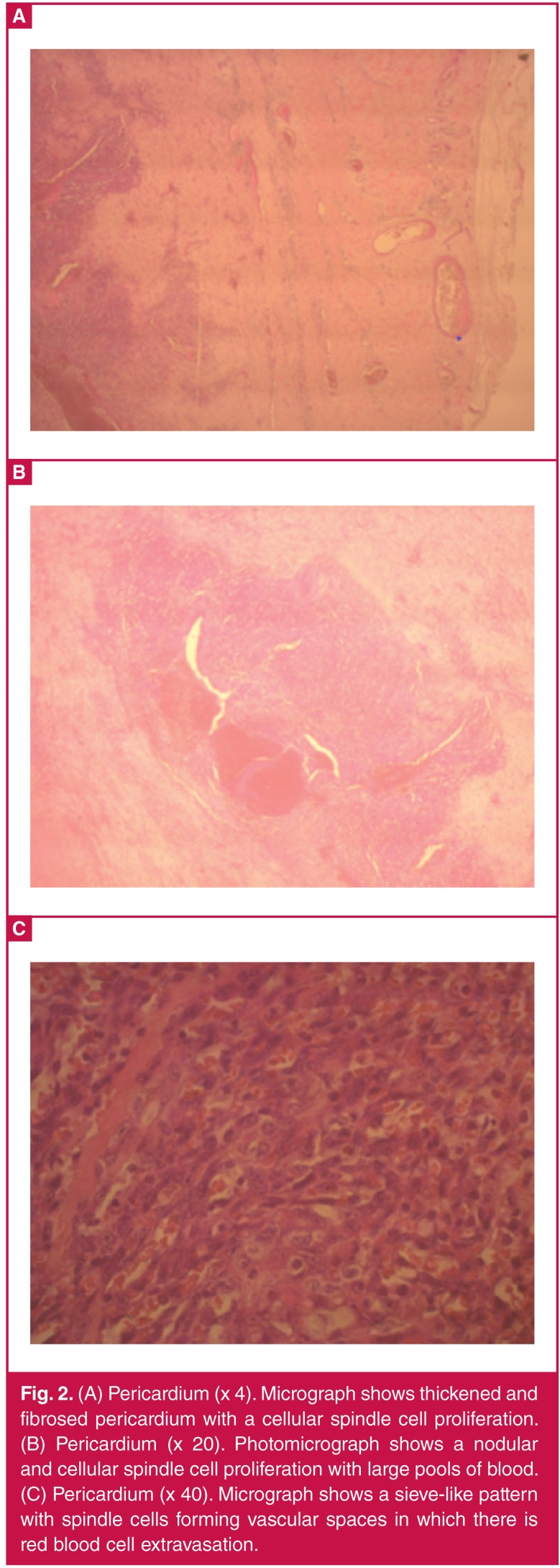
(A) Pericardium (x 4). Micrograph shows thickened and fibrosed pericardium with a cellular spindle cell proliferation. (B) Pericardium (x 20). Photomicrograph shows a nodular and cellular spindle cell proliferation with large pools of blood. (C) Pericardium (x 40). Micrograph shows a sieve-like pattern with spindle cells forming vascular spaces in which there is red blood cell extravasation.

A pericardial biopsy showed a cellular tumour comprising vasoformative spindle cells with extravasation of red blood cells and eosinophilic bodies. Moderate nuclear pleomorphism and a brisk mitotic count were seen. This was consistent with an angiosarcoma (Fig. 2).

The patient was referred to oncology care after debulking and he received a pulse of chemotherapy (vincristine, doxorubicin and cyclophosphamide). He became paraplegic and eventually died five months after his initial presentation.

## Discussion

Primary cardiac tumours are rare and angiosarcoma is the most frequent primary malignant cardiac tumour.^6^ Angiosarcomas of the pericardium are rare but there have been several case reports.^7^-^10^ They usually occur in the third to the fifth decade of life and are more common in males. By the time these tumours are diagnosed, 66 to 89% have metastases. They have a poor prognosis. The mean survival is six to 11 months. Metastatic disease is frequent at the time of presentation, mainly to the mediastinal lymph nodes, lung and vertebrae. Our patient’s tumour was quite invasive and had metastases to several vertebrae.

In this patient, the initial radiological investigation was an echocardiogram, which revealed a fibrinous pericardial effusion. Echocardiography is usually the initial diagnostic tool for cardiac tumours. Transthoracic and transoesophageal echocardiography have a sensitivity of 93 and 97%, respectively, for detecting cardiac masses.^11^ These however are operator and technique dependant.

CT and magnetic resonance imaging reveal more detail in terms of cardiac soft tissue as well as extracardiac extension of cardiac tumours. In this patient it was the transthoracic echocardiogram at six weeks, followed by the CT scan that suggested a malignant cause for the pericardial effusion.

Pericardial fluid cytology is unreliable and is not diagnostic in the majority of patients. Pericardial or endomyocardial biopsy will be diagnostic in 23 to 50% of samples.^11^ The microscopy of cardiac angiosarcoma is characterised by anastomotic vascular channels formed by malignant cells, solid areas of spindle cells and areas of anaplastic cells. This patient’s biopsy specimens showed the typical histological features.

This case illustrates the challenges of making a definitive diagnosis of TB pericarditis in resource-poor settings and that clinical index can be found wanting when faced with alternative pathology, as in this patient.

The definitive diagnosis of TB pericarditis is known to be challenging. The symptoms, chest pain, shortness of breath, fever and night sweats, are not specific. The signs of a large effusion include a small-volume pulse, raised jugular venous pulsations, diffuse apex beat, muffled heart sounds and hepatomegaly. The presence of fever and a supraclavicular lymph node makes TB a most probable cause of the effusion. Chest X-ray, ECG and echocardiography are not specific for TB. There are other causes of fibrinous pericardial effusions, such as viral and bacterial infections, uraemia and malignancy.

The definitive diagnosis hinges on finding mycobacteria on pericardial fluid microscopy or culture as well as histological examination of a pericardial biopsy. Finding mycobacteria in other specimens, such as sputum, gastric washings and pleural fluid in a patient with a fibrinous pericardial effusion makes TB the most likely cause of the effusion.^12^

However, direct smear is only positive in 0–42% of cases of TB pericarditis. Conventional culture is positive in up to 53% and this can be improved if direct culture onto liquid Kirchner culture medium is done. The rate of positive culture goes up to 75%.

In resource-poor settings, microbiology services are limited so both direct smear and culture are not always available. The other problem with TB culture is the long delay in getting the results and for a condition where immediate therapy is needed, treatment is usually commenced before these results are available. Pericardial biopsy is invasive and requires the expertise of a surgeon and this is not usually available where TB is most common. Pericardial biopsy is diagnostic in 10–64% of cases.^7^

Other methods to make a diagnosis of probable pericardial TB include finding a lymphocyte predominance and a high protein level in the fluid, clinical index (Tygerberg score), PCR and indirect tests such as ADA, lysozyme and IFN gamma. The Tygerberg score comprises weight loss = 1, night sweats = 1, fever of ≥ 38°C = 2, peripheral white cell count < 10 cells/μl = 3, and serum globulin > 40 g/l = 3. A total score ≥ 6 has a sensitivity of 86% and a specificity of 85%. This is a reasonable approach in a resource-poor setting.

PCR tends to be expensive, unavailable, and has a high rate of false-positive results. Adenine deaminase > 40 IU/l has a sensitivity of 87% and a specificity of 83%, IFN gamma > 50 pg/l has a sensitivity of 92% and specificity of 100% and lysozyme > 6.6 μg/dl has 100% sensitivity and 91% specificity. These three tests are very good but cost and availability are the limiting factors in sub-Saharan Africa where TB is very common.

This patient had a low ADA of 24 IU/l; an ADA of > 35 IU/l has a sensitivity of > 95%.^13^ The low ADA should have been used much earlier as a rule-out test for TB pericarditis and an alternative cause could have been sought earlier.

In most cases that are treated as TB pericarditis in sub-Saharan Africa, the clinical criteria are used to make a decision to treat. In the majority of patients, this is the correct decision, but it is that occasional patient such as the one described, where the special tests to make a definitive diagnosis would have clinched the alternative diagnosis earlier.

This patient illustrates the need to be aware of the rarer causes of fibrinous pericardial effusion and the need to perform more tests, such as CT or MRI scans and pericardial biopsy to make a definitive diagnosis, even in our setting of high TB prevalence. Unfortunately however, most cases of angiosarcoma present with metastatic deposits and the options for therapy may not be available, as it was for this patient. The prognosis tends to be poor.
